# Impact of SOD1 Transcript Variants on Amyotrophic Lateral Sclerosis Severity

**DOI:** 10.3390/ijms26146788

**Published:** 2025-07-15

**Authors:** Matteo Bordoni, Eveljn Scarian, Camilla Viola, Francesca Dragoni, Rosalinda Di Gerlando, Bartolo Rizzo, Luca Diamanti, Stella Gagliardi, Orietta Pansarasa

**Affiliations:** 1 Cellular Models and Neuroepigenetics Section, IRCCS Mondino Foundation, Via Mondino 2, 27100 Pavia, Italy; matteo.bordoni@mondino.it (M.B.); eveljn.scarian@mondino.it (E.S.); camilla.viola@mondino.it (C.V.); orietta.pansarasa@mondino.it (O.P.); 2 Department of Brain and Behavioral Sciences, University of Pavia, Via Agostino Bassi 21, 27100 Pavia, Italy; 3 Molecular Biology and Transcriptomics Section, IRCCS Mondino Foundation, Via Mondino 2, 27100 Pavia, Italy; francesca.dragoni@mondino.it (F.D.); rosalinda.digerlando@mondino.it (R.D.G.); 4 Department of Biology and Biotechnology “L. Spallanzani”, University of Pavia, Via Adolfo Ferrata 9, 27100 Pavia, Italy; 5 Golgi Cenci Foundation, Corso S. Martino 10, 20081 Abbiategrasso, Italy; b.rizzo@golgicenci.it; 6 Neuroncology and Neuroinflammation Unit, IRCCS Mondino Foundation, Via Mondino 2, 27100 Pavia, Italy; luca.diamanti@mondino.it

**Keywords:** amyotrophic lateral sclerosis, *SOD1*, transcript, oxidative stress, disease severity

## Abstract

Amyotrophic lateral sclerosis (ALS) is an incurable neurodegenerative disease that affects motor neurons of people, leading to death. This pathology can be caused by mutations in different genes, including superoxide dismutase 1 (*SOD1*). Previous studies have pointed out the presence of two transcripts of *SOD1*, a short one and a long one. The aim of this study was the investigation of these two transcripts both in the SH-SY5Y cell line and in patients’ peripheral blood mononuclear cells. We found that the shortest *SOD1* transcript is upregulated under stress conditions in both the cellular model and the patients’ cells. Moreover, we found a potential correlation between the short *SOD1* transcript and the severity of the pathology, which also correlates with the age of patients. No correlation was found between *SOD1* transcripts and the progression of the disease. These data suggest a toxic effect of short *SOD1* transcripts in ALS patients, by affecting the severity of the pathology making it a possible biomarker for this disease. Interestingly, our data suggest that a short *SOD1* transcript does not influence and drive disease progression. The finding of a biomarker will have suitable implications as indicators of disease severity and from the perspective of drug development.

## 1. Introduction

Amyotrophic lateral sclerosis (ALS) (OMIM ID: 105400) is a fatal and progressive neurodegenerative disease that damages motor neurons. It is considered a rare disease since about 2 people in 100,000 are affected [1]. The pathology causes the death of upper and lower motor neurons in the motor cortex, brainstem, and spinal cord, leading to death usually by respiratory failure [2]. Traditionally, ALS has been divided into a ‘sporadic’ form (sALS), which represent 90% of cases, and a ‘familial’ form, 10% of cases, and there is evidence of mutations in apparently sporadic cases [3]. More than 30 genes have been implicated in genetic forms of ALS, but the most common mutated genes are *C9ORF72*, superoxide dismutase 1 (*SOD1*), *TARDBP*, and *FUS* [4,5].

The pathogenic mechanism that leads to motor neuron degeneration in ALS is not completely clear. Several cellular and molecular processes have been found to be implicated, e.g., mitochondrial dysfunction, toxic protein aggregation, impaired protein degradation, prion-like spreading, excitotoxicity, oxidative stress (OS), RNA metabolism defects, and RNA toxicity [6].

In 1993, Rosen and colleagues linked for the first time mutations in *SOD1* to ALS [7]. *SOD1* was the first ALS gene identified, and it is present in approximately 8–23% of familial ALS and 1–4% of sALS cases [8]. Familial ALS cases associated with *SOD1* mutations are designated as ALS1 [9]. The gene is formed of five exons and four intronic regions and possesses several poly(A) signal sequences on the 3′ end [7,10].

More than 200 disease-associated *SOD1* mutations, most of which are missense, have been identified, and they can be found virtually in every region of the 153-amino acid SOD1 polypeptide [11]. Moreover, there is a huge phenotypic heterogeneity amongst patients with the same mutation, indicating that epigenetic and environmental factors could influence disease expression [12,13].

Mutations usually lead to low enzyme activity, OS, since the enzyme is normally involved in reactive oxygen species elimination, endoplasmic reticulum, and mitochondrial dysfunction [12,14]. Like other genes involved in ALS pathology, mutated *SOD1* is associated with protein re-localization and aggregation [15,16]. Furthermore, a prion-like spreading of SOD1 was repeatedly demonstrated [17,18,19]. Curiously, *SOD1*-linked ALS lacks TDP43 inclusions, considered a hallmark for most ALS cases [20,21].

In 1984, Sherman and colleagues identified for the first time two different *SOD1* transcripts that differ in their polyadenylation signals at 3′UTR: one mRNA of 0.7 kb is approximately four time more abundant than the other one of 0.9 kb [11,12]. Moreover, it was demonstrated that the 3′UTR region of the 0.9 kb *SOD1* transcript presents five adenylate/urydilate-rich elements, which can act as binding sites for proteins, stabilizing the transcript [13,14]. Adenylate/urydilate-rich elements bind transcription factors such as ELAVL4 (HuD), a member of the ELAVL family, required for neuronal differentiation and strongly associated with neurodegenerative diseases [15]. Precedent studies have also demonstrated an involvement of HuD in ALS pathogenesis, inasmuch as it interacts with mRNAs of FUS [16]. Thus, the aim of this work was the investigation of the biological role of both short (0.7 kb) and long (0.9 kb) transcripts of *SOD1*. We evaluated the level of these two variants in both sALS patients and in cellular models and their possible effects. Finally, we correlate their levels with several patients’ characteristics.

## 2. Results

### 2.1. Oxidative Stress Induces the Overexpression of SHORT SOD1 Transcript Leading to the Increase and Aggregation of TOT SOD1

**Figure 1:**
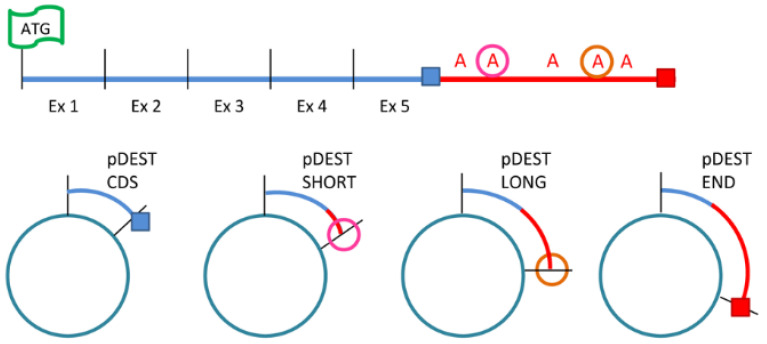
Schematic representation of the plasmids. The four plasmids contained four *SOD1* transcript sequences differing for the 3′UTR length. The coding sequence only, which ends with the stop codon (blue square) (pDEST CDS); the SHORT *SOD1* transcript, which ends with the 2nd polyA signal (pink circle) (pDEST SHORT); the LONG *SOD1* transcript, which ends with the 4th polyA site (orange circle) (pDEST LONG); and the *SOD1* transcript containing the entire 3′UTR (red square) (pEST END).

Two *SOD1* transcripts differing in the length of their 3′UTRs, 0.7 kb and 0.9 kb, were first described by Sherman in 1984 [22]. In this work, to better understand the role of these two transcripts, we cloned the long (LONG *SOD1*) and the short (SHORT *SOD1*) transcripts into pDEST30 plasmid and we generated four *SOD1* plasmids fused with an N’terminal-Flag sequence (FLAG): the coding sequence only (which ends with the stop codon) (blue square, CDS *SOD1*), a SHORT *SOD1* transcript that ends with the 2nd polyA signal (pink circle, pDEST SHORT), a LONG *SOD1* transcript that terminates with the 4th polyA site (orange circle, pDEST LONG), and a *SOD1* transcript containing the entire 3′UTR (terminating with the red square, END *SOD1*) (Figure 1).

We then transfected these plasmids into both HeLa and SH-SY5Y cells, as non-neural and neural cellular models, respectively. We confirmed the correct transfection in HeLa cells (Figure S1A,B); however, in both transfected and not-transfected (NT) HeLa cells, we did not find any differences in the expression of endogenous *SOD1* (Figure S1C,D). These data were confirmed by immunofluorescence (IF) (Figure S1E–H).

**Figure 2:**
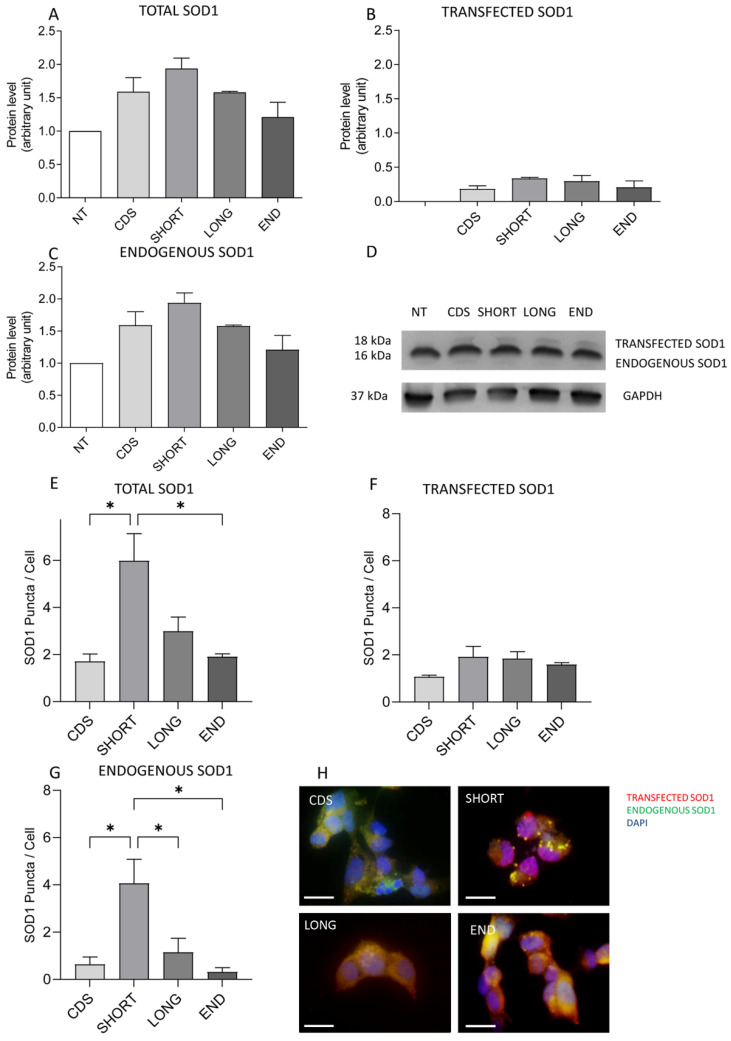
Total and endogenous *SOD1* is increased in SHORT SOD1 transfected SH-SY5Y cells. Four SOD1 plasmids were generated and transfected in SH-SY5Y cells: CDS *SOD1* (coding sequence ending with the stop codon), SHORT *SOD1* (transcript ending with the 2nd polyA signal), LONG *SOD1* (transcript ending with the 4th polyA site), and END *SOD1* (transcript ending with the 4th polyA site). NT indicates non-transfected SH-SY5Y cells. In the figure, (**A**–**D**) show the WB analysis results for total SOD1, transfected SOD1, and endogenous SOD1. (**A**) A not significant increase was found in the expression of total SOD1 in SHORT SOD1 transfected cells compared to the other transcripts. (**B**) A not significant increase was found in transfected SOD1 protein expression. (**C**) A not significant increase was found in the expression of endogenous SOD1 in SHORT SOD1 transfected cells. (**D**) shows a representative WB of endogenous (16 kDa) and transfected (18 kDa) protein levels. GAPDH was used as the loading control (37 kDa). In the figure, (**E**–**H**) show the IF analysis results for total *SOD1*, transfected *SOD1*, and endogenous *SOD1*. (**E**) A significant increase in endogenous *SOD1* was found in SHORT *SOD1* transfected cells compared to CDS (* *p* = 0.0120) and END *SOD1* (* *p* = 0.0133). (**F**) No significant differences were found for transfected *SOD1*. (**G**) A significant increase in endogenous *SOD1* was also found in SHORT *SOD1* transfected cells compared to CDS (* *p* = 0.0215), LONG (* *p* = 0.0401), and END *SOD1* (* *p* = 0.0157). (**H**) Representative images of IF analysis (red= transfected *SOD1*, green= endogenous *SOD1*, blue= nuclei stained by DAPI). Scale bar: 10µm. Error bars indicate the standard error of the mean (S.E.M.). N = 3. Data were analyzed using an ANOVA test followed by Bonferroni’s test.

Thus, we focused on the neural cellular model, i.e., SH-SY5Y cells. In a previous work, we demonstrated that SH-SY5Y cells are a good model with which to study mechanisms underlying neurodegenerative diseases. In particular, both the treatments with hydrogen peroxide (H_2_O_2_) [23,24] and the transfection with disease-related plasmids [25] mimic some features of ALS pathomechanisms. We firstly confirmed the successful transfection in SH-SY5Y cells (Figure 2A,B). Using Western blot (WB) analysis, we found an increasing trend in the expression of both total and endogenous SOD1 in SHORT SOD1 transfected cells compared to the other variants (Figure 2A,C). On the contrary, no differences were found in transfected SOD1 (Figure 2B). By IF analysis, we investigated the presence of pathological SOD1 aggregation. We found a statistically significant increase in the total SOD1 puncta in SHORT SOD1 transfected cells compared to CDS (* *p* = 0.0120) and to END SOD1 (* *p* = 0.0133) (Figure 2E). A statistically significant increase in the endogenous SOD1 puncta in SHORT SOD1 transfected cells compared to CDS (* *p* = 0.0215), LONG (* *p* = 0.0401), and END (* *p* = 0.0157) SOD1 was also reported (Figure 2G).

**Figure 3:**
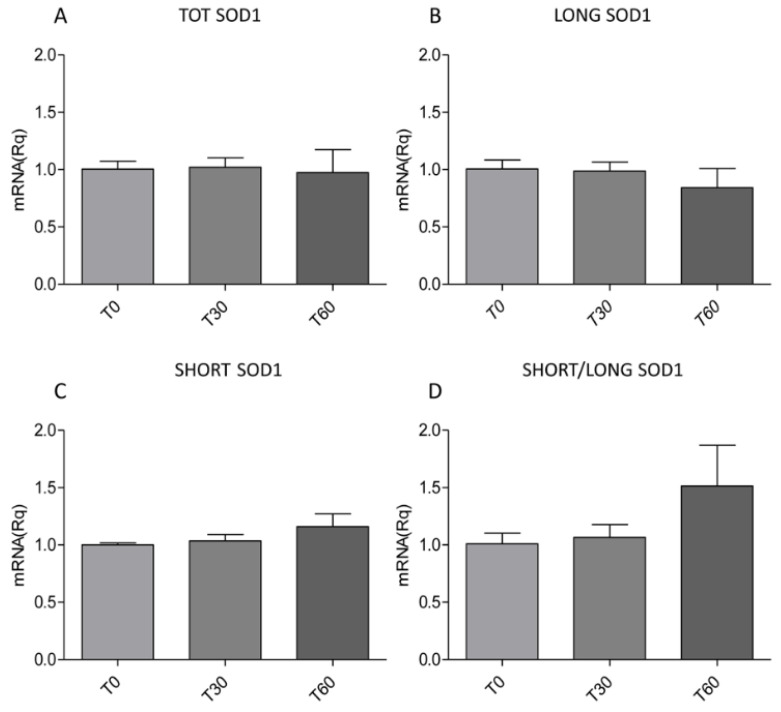
Oxidative stress treatment does not influence the levels of TOT *SOD1*, LONG *SOD1*, and SHORT *SOD1*. The levels of TOT *SOD1*, LONG *SOD1*, and SHORT *SOD1* in SH-SY5Y were evaluated by multiplex RT-PCR at T0 (non-treated condition), T30 (after 30 min of 1 mM H_2_O_2_ treatment), and T60 (after 60 min of 1 mM H_2_O_2_ treatment). (**A**–**C**) No significant differences were found in the levels of TOT *SOD1*, LONG *SOD1*, and SHORT *SOD1*. (**D**) A not significant increase was found in the ratio of SHORT *SOD1* to LONG *SOD1* after 60 min of 1 mM H_2_O_2_ treatment. Error bars indicate S.E.M. N = 3. Data were analyzed using an ANOVA test followed by the Bonferroni test.

All these data suggest a role of SHORT *SOD1* in SOD1 aggregation and greater specificity of the SH-SY5Y neuronal model. In particular, since we found an increase in SOD1 aggregation when we induce SHORT SOD1 protein expression, we hypothesize a toxic function of this form. To assess this hypothesis, we used 1 mM H_2_O_2_-treated SH-SY5Y cells, a well-characterized cellular model that mimics several pathological features of ALS [23,24,25]. We analyzed the levels of total *SOD1* (TOT *SOD1*), LONG *SOD1*, and SHORT *SOD1* (Figure 3A–C) by multiplex reverse transcription–polymerase chain reaction (RT-PCR), and we did not find any differences after OS induction (T30 and T60). Notwithstanding, when we looked at the ratio of SHORT *SOD1* to LONG *SOD1*, we observed an interesting, but not significant, increase in this ratio after prolonged OS induction (T60) (Figure 3D).

### 2.2. Peripheral Blood Mononuclear Cells of sALS Patients Present an Increased Expression of SHORT SOD1

In 2016, we first reported that SH-5YSY cells treated with 1 mM H_2_O_2_ for 60 min display pathological features typical of sALS patients [24]. Here, we further proved that also transfected SH-SY5Y cells treated with H_2_O_2_ at the same concentration and for the same time are a valid ALS model. Based on this in vitro evidence, we decided to evaluate if the differences found in SH-SY5Y cells, regarding LONG and SHORT *SOD1*, could be detected in peripheral blood mononuclear cells (PBMCs) derived from sALS patients.

**Figure 4:**
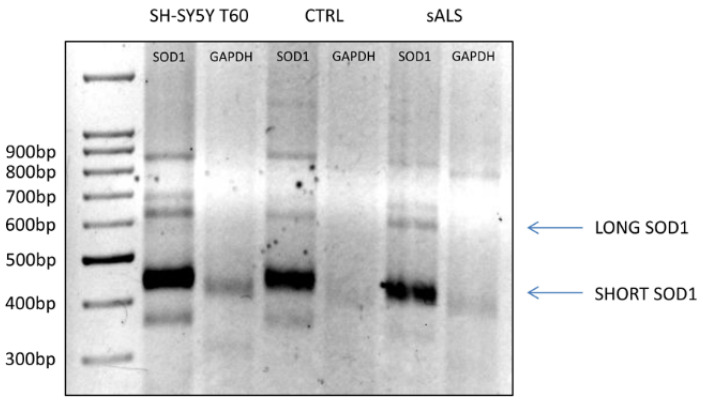
sALS patients’ peripheral blood mononuclear cells express less LONG *SOD1* and more SHORT *SOD1*. 3′RACE was performed on the PBMCs of sALS patients and the CTRL. SH-SY5Y cells exposed to 1 mM H_2_O_2_ for 60 min (T60) were used as a positive control. *GAPDH* was used as a negative control to confirm the presence of different isoforms amplified through 3′RACE. A SHORT *SOD1* amplicon had a length of about 400 bp, while a LONG *SOD1* amplicon had a length of about 600 bp. CTRL and sALS PBMCs expressed both LONG and SHORT *SOD1*. sALS patients showed a lower expression of LONG *SOD1* and a higher expression of SHORT *SOD1* compared to those of the CTRL.

To evaluate the presence of both SHORT *SOD1* and LONG *SOD1* transcripts in patients, we performed a 3′ rapid amplification of cDNA ends (RACE) on PBMCs of sALS patients and healthy controls (CTRL) (Figure 4). As a positive control, we used SH-SY5Y cells exposed to OS for 60 min (T60), while *GAPDH* was used as a negative control. By means of 3′RACE, we confirmed the presence of *SOD1* transcripts (LONG and SHORT *SOD1*) also in the PBMCs of sALS patients and the CTRL. In particular, sALS patients have a lower expression of LONG *SOD1* and a higher expression of SHORT *SOD1* (Figure 4).

**Figure 5:**
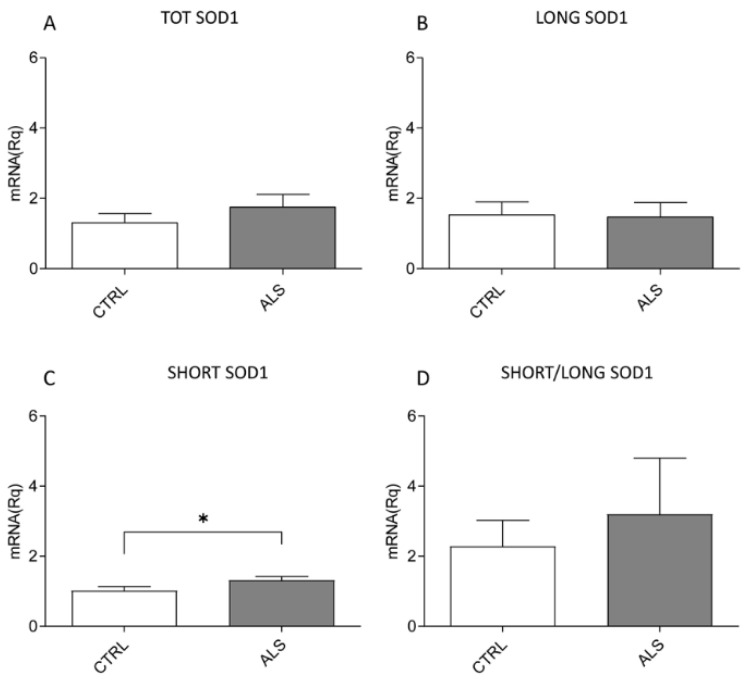
sALS peripheral blood mononuclear cells (PBMCs) have a higher expression of SHORT *SOD1* than CTRL ones. The levels of TOT *SOD1*, LONG *SOD1*, and SHORT *SOD1* transcripts in sALS patients and CTRL PBMCs were evaluated by multiplex RT-PCR. (**A**) An increase, although not significant, was found in TOT *SOD1* expression in sALS PBMCs when compared to CTRL PBMCs. (**B**) No changes in LONG *SOD1* expression were found. (**C**) A significant increase (* *p* = 0.0429) was found in SHORT *SOD1* expression in sALS PBMCs compared to CTRL PBMCs. (**D**) The ratio between SHORT and LONG transcript expression was calculated, finding a non-significant increase in ALS PBMCs (**D**). Error bars indicate S.E.M. N = 15. Data were analyzed using an unpaired one-tailed *t*-test.

Since 3′RACE is not a quantitative analysis, we also performed multiplex RT-PCR to evaluate and quantify the level of each transcript (Figure 5). We found an increase, although not significant, in the TOT *SOD1* expression in sALS patients compared to in CTRL subjects (Figure 5A). When we separately analyzed the levels of LONG and SHORT *SOD1*, we found no change in LONG *SOD1* expression (Figure 5B) and a significant increase in SHORT *SOD1* (* *p* = 0.0429) (Figure 5C) in sALS patients. Similarly to previous results in SH-SY5Y cells after OS induction (T60), by analyzing the ratio between SHORT and LONG *SOD1* values in both CTRL and sALS PBMCs, we found a non-significant increase in the SHORT to LONG *SOD1* ratio in sALS patients (Figure 5D). These data further address a different role of the two *SOD1* transcripts in ALS’ pathomechanism.

### 2.3. LONG SOD1 Inversely Correlates with the Severity of the Pathology

We thus correlate multiplex RT-PCR data with some of the subjects’ clinical features to point out the possible role of *SOD1* transcript in ALS pathology.

**Figure 6:**
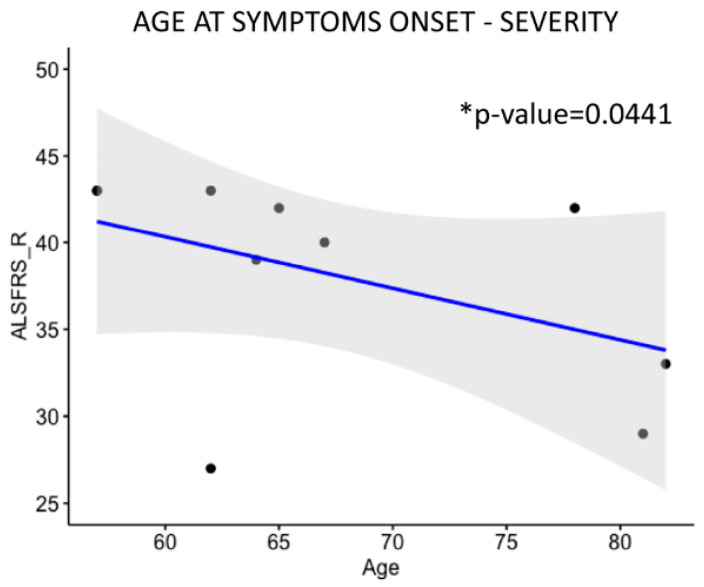
Patients’ age at symptom onset is negatively correlated with ALSFRS_R values. Patients’ ages at the onset of symptoms were correlated with the ALSFRS_R values. A significant negative correlation was found (* *p* = 0.0441). Data were analyzed using a one-tailed Spearman test. N = 10.

**Figure 7:**
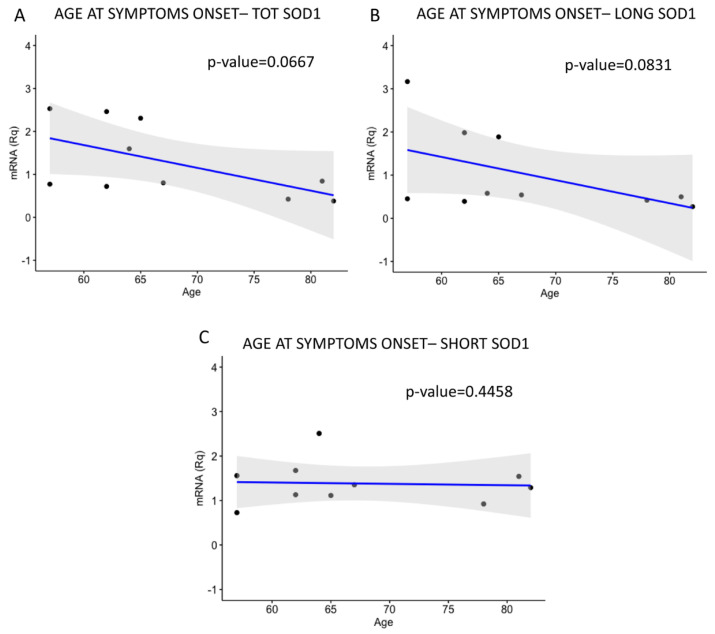
Patients’ age at symptom onset is not correlated with *SOD1* transcript expression. The levels of TOT *SOD1*, LONG *SOD1*, and SHORT *SOD1* were correlated with the ages of patients at symptom onset. (**A**,**B**) No significant negative correlations between both TOT *SOD1* and LONG *SOD1* and patients’ ages at symptom onset were found. (**C**) No significant differences were found between SHORT *SOD1* and ages at symptom onset. Data were analyzed using a one-tailed Spearman test. N = 10.

**Figure 8:**
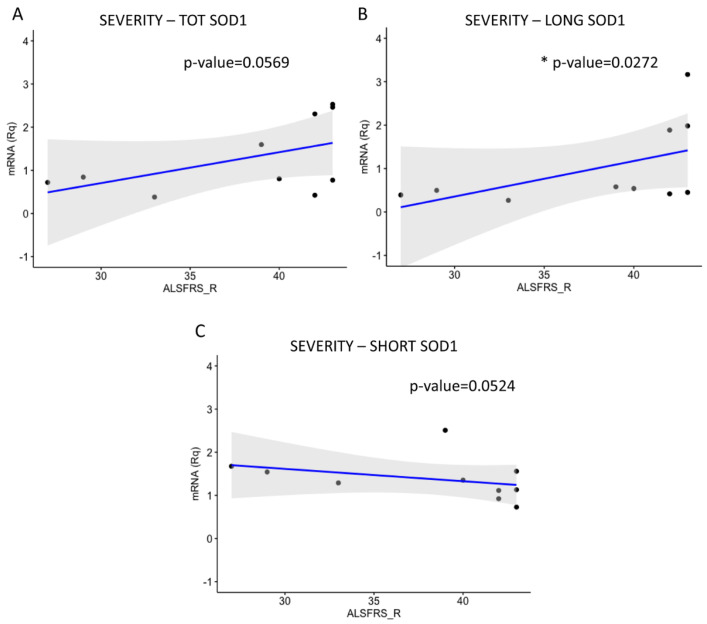
ALSFRS_R value correlates with LONG *SOD1* expression. The levels of TOT *SOD1*, LONG *SOD1*, and SHORT *SOD1* were correlated with the ALSFRS_R values. (**A**) No significant positive correlation between TOT *SOD1* and ALSFRS_R values was found (*p* = 0.0569). (**B**) A significant positive correlation between LONG *SOD1* and ALSFRS_R was detected (* *p* = 0.0272). (**C**) No statistically significant negative correlation was found between SHORT *SOD1* and ALSFRS_R values (*p* = 0.0524). Data were analyzed using a one-tailed Spearman test. N = 10.

Patients’ ages at symptom onset were first correlated with the severity of the pathology, calculated through the ALS Functional Rating Scale_Revised (ALSFRS_R) value (Figure 6) and then with TOT *SOD1*, LONG *SOD1*, and SHORT *SOD1* levels (Figure 7). Finally, transcripts levels were correlated with the ALSFRS_R value (Figure 8), basal progression rate (PRB) (Figure S2), and late progression rate (PRL) (Figure S3).

As shown in Figure 6, we found a negative correlation between the ALSFRS_R value and patients’ age at disease onset (* *p* < 0.0441), indicating a positive correlation of patients’ age and the loss of physical function over time. Thus, we decided to investigate a possible correlation between *SOD1* transcripts levels and both the patient’s age and ALSFRS_R value.

We found a negative correlation between patients’ ages at symptom onset and both TOT *SOD1* (Figure 7A) and LONG *SOD1* values (Figure 7B), although without significant differences. We did not find any correlation with SHORT *SOD1* (Figure 7C).

As regards the severity of the pathology, we found a positive correlation between TOT *SOD1* and the ALSFRS_R value, with a *p*-value near the statistical significance (Figure 8A). A positive correlation was also found between LONG *SOD1* and the ALSFRS_R value (* *p* < 0.0272) (Figure 8B). We found a negative correlation with SHORT *SOD1*, although without a significant difference (Figure 8C). These data suggest that the positive correlation of TOT *SOD1* with the ALSFRS_R value is mainly due to LONG *SOD1*. Moreover, patients with a higher value of SHORT *SOD1* manifested a lower value of ALSFRS_R, indicating a possible correlation between SHORT *SOD1* levels and ALS severity. These data suggest that patients’ age is correlated with a decrease in physical function and with lower values of TOT *SOD1* and LONG *SOD1*.

Finally, we investigated possible correlations between TOT *SOD1*, LONG *SOD1*, and SHORT *SOD1* values and PRB (Figure S2) and PRL (Figure S3). With regard to PRB as well as PRL, we did not find any significant correlation.

## 3. Discussion

In 1984, Sherman and colleagues described for the first time two *SOD1* transcripts of about 0.7 and 0.9 kb. The authors found that the two transcripts differ in the length of their 3′-UTR; in particular, the long transcript contains 222 additional nucleotides beyond the 3′-polyadenylated terminus of the short mRNA [22]. Moreover, the 0.7 kb transcript is approximately four times more abundant than the 0.9 kb one [22]. Since *SOD1* is known to have a pivotal role in ALS pathogenesis [15], here, we investigated for the first time the biological role of the two different transcripts of *SOD1* in cellular models and in the PBMCs of sALS patients. We used both non-neural (HeLa) and neural (SH-SY5Y) cellular models to investigate whether the two transcripts of SOD1 act differently in the two tissues.

SH-SY5Y cells, after the induction of OS through 1 mM H_2_O_2_ treatment, closely mimic the molecular underpinnings characteristic of ALS [23,24,25]. Thus, they can be used as a model to test specific disease pathways and the function of ALS-related proteins and genes in a more convenient and standardized manner. Our findings point toward a change in total *SOD1* mainly due to the increased expression of SHORT *SOD1*. In particular, by IF analysis, we found an increase in aggregated SOD1, as suggested by the puncta analysis, supporting the hypothesis that SHORT *SOD1* expression has toxic and pathogenic functions. Interestingly, we found an increase in SHORT SOD1 in the PBMCs of sALS patients. However, the relevance and meaning of our findings on the SHORT *SOD1* transcript in SH-SY5Y cells and in the PBMCs of sALS patients are challenging to interpret. Our guess is that they may be due to a shift in mRNA transcription under OS conditions as well as in sALS patients. Since we found aggregation of SOD1, we hypothesize that the SHORT *SOD1* transcript generates a protein more susceptible to misfolding compared to the other transcript variants. In turn, the misfolded SOD1 leads to the amplification of aggregation in a prion-like manner [26,27].

We thus correlated multiplex data with some of the subjects’ clinical features to point out the possible role of *SOD1* transcripts in ALS pathology. The multiplex RT-PCR results on sALS PBMCs were further investigated to pinpoint a possible correlation with patients’ age at symptom onset and with clinical scores. Interestingly, a negative correlation was found between LONG SOD1 and pathology severity, suggesting an imbalance of this form in pathological conditions, probably caused by reactive oxygen species damage as observed in ALS [15,28,29]. Additionally, several authors correlate mutations in *SOD1* with a major pathology acuteness, indicating a pivotal role of this gene in ALS [30,31]. Moreover, our data indicate a more severe pathology in older patients at symptom onset, suggesting that the exacerbation of clinical symptoms is probably due to a reduction in TOT *SOD1* and in the long transcript, and to an increase in the short one. To our knowledge, no previous studies have analyzed the correlation between symptom onset age and the severity of ALS pathology.

Although recent advances have clarified a more objective measure of disease progression, the phenotypic variability as well as the non-linearity during the disease course keep complicating the measurement of functional decline in ALS patients [32].

In this study, we pointed out the possible role of *SOD1* variants in ALS pathology. However, this study suffers from some issues. The analysis only included a small number of sALS patients (N = 15) and healthy participants (N = 12). Additionally, the number of sALS patients was reduced for the correlation analysis to N = 10. This reduction was due to the fact that not all the patients returned for a follow-up appointment, either as a result of the worsening of their pathology, making it impossible for them to move, or because they passed away. To determine the PRL value, it is known that a second visit is necessary. For this reason, we decided to exclude patients without the second visit in all correlation analyses. The small sample size of ALS patients limits the statistical power; consequently, new studies are needed to confirm these data on a larger cohort of patients. Because of the small sample size and the high variability of ALS patients, our work is speculative, but the statistical significance found on the correlation with the severity, suggest that this research is a good starting point for further multicentric studies with a larger cohort. Likewise, the presence of outlier data, particularly in the correlation analysis, could provide additional evidence of the absence of significance and, more remarkably, of the phenotypic variability among ALS patients.

Furthermore, the SHORT SOD1 isoform appears to be upregulated in ALS. This expression pattern might reflect a fetal-like reactivation rather than a disease-specific mechanism. Unfortunately, we did not include fetal samples in our analysis, and no data are currently available to determine whether this isoform is expressed during human development. Future studies including fetal and developmental-stage tissues will be necessary to clarify this point.

Finally, a last limitation could be the low transfection efficacy of SH-SY5Y cells. This weakness could account for the lack of significance of some results.

## 4. Materials and Methods

### 4.1. HeLa and SH-SY5Y Cell Culture and Treatment

Cervical cancer HeLa cells and neuroblastoma SH-SY5Y cells were cultured in Dulbecco’s Modified Eagle’s Low Glucose Medium (Euroclone, Pero, Milan, Italy) supplemented with 15% Fetal Bovine Serum, 2 mM L-glutamine 1% streptomycin (100 U/mL; 100 mg/mL), and 1% penicillin (100 U/mL; 100 mg/mL) (all supplied by Carlo Erba, Cornaredo, Milan, Italy). Cells were grown in a humidified atmosphere containing 5% CO_2_ and at 37 °C.

HeLa and SH-SY5Y cells were treated with 1 mM H_2_O_2_ (Sigma-Aldrich, St. Louis, MO, USA) for the induction of OS [24,25]. Cells were exposed to 1X phosphate-buffered saline solution (Sigma-Aldrich, St. Louis, MO, USA) for the non-treated condition (T0) or treated with 1 mM H_2_O_2_ for 30 (T30) or 60 (T60) min. Cells were washed with 1X phosphate-buffered saline solution and centrifuged for the formation of pellets, and then conserved at −80 °C until further use. Each treatment was performed three times and when cells reached a confluence of 80–90%.

### 4.2. Enrolment of sALS Patients and Healthy Subjects

PBMCs were isolated from 15 sALS patients (eight males and seven females, mean age= 67.3 ± 7.4) after 6–9 months from disease onset. PBMCs were also isolated from 12 sex- and age-matched healthy CTRL subjects (six males and six females, mean age = 62.8 ± 4.6). sALS patients were recruited and diagnosed at IRCCS Mondino Foundation (Pavia, Italy) according to El Escorial Criteria [33]. Participants were tested for genetic mutations by Next-Generation Sequencing (Illumina, San Diego, CA, USA) as previously described [34]. A correlation was made on N = 10 sALS patients, and their clinical characterization is reported in Table S1. CTRL subjects were recruited at IRCCS Policlinico S. Matteo Foundation (Pavia, Italy) and interviewed for personal history to avoid the presence of any chronic or neurodegenerative disease, and to exclude familiarity with any motor neuron disease.

### 4.3. Peripheral Blood Mononuclear Cells’ Isolation

Total blood was obtained from sALS patients and CTRL subjects through venipuncture and conserved into Ethylenediaminetetraacetic acid tubes to avoid coagulation. PBMCs were isolated within 24 h using Ficoll-Histopaque^®^-1077 (Sigma-Aldrich, St. Louis, MO, USA) following the manufacturer’s instructions. Cell viability was evaluated through the Trypan Blue (Sigma-Aldrich, St. Louis, MO, USA) Exclusion Test using an automatic cell counter (TC20 Automated Cell Counter, BioRad, Hercules, CA, USA). Aliquots of PBMCs from both sALS patients and CTRL subjects were cryopreserved in Fetal Bovine Serum (EuroClone, Pero, Milan, Italy) and dimethyl sulfoxide (Sigma-Aldrich, St. Louis, MO, USA) to avoid cell death. PBMCs were stored at −80 °C for 24 h and then in liquid nitrogen.

### 4.4. Cell Transfection

In 1984, Sherman identified two *SOD1* mRNA from the same gene, SHORT *SOD1* and LONG *SOD1*, which differ in the length of their 3′-UTR regions (Figure S4). We generated four *SOD1* plasmids (CDS, SHORT, LONG, and END) fused with FLAG. Plasmids were generated using Gateway technology (Invitrogen, Waltham, MA, USA) following the manufacturer’s instructions. HeLa and SH-SY5Y cells were transfected using Lipofectamine 2000 (Life Technologies, Carlsbad, CA, USA) following the manufacturer’s instructions. We then measured, via IF and WB, both endogenous and transfected *SOD1* using, respectively, anti-*SOD1* and anti-FLAG antibodies.

### 4.5. Immunofluorescence Analysis

The fixation of 3 × 10^5^ transfected HeLa and SH-SY5Y cells was performed in 4% paraformaldehyde (ThermoFisher Scientific, Waltham, MA, USA). Cells were then blocked in 5% Normal Goat Serum (ThermoFisher Scientific, Waltham, MA, USA) and 0.1% Tween (Sigma-Aldrich, St. Louis, MO, USA) and incubated in primary antibodies at 4 °C overnight (Table S2). The next day, cells were incubated in secondary antibodies at room temperature for 1 h (Table S2), mounted with Prolong^®^Gold anti-fade reagent DAPI (Invitrogen, Waltham, MA, USA), dried, and nail-polished. Images were acquired using an Axio Imager 2 fluorescence microscope (Zeiss, Hebron, KY, USA), and analysis was performed using ImageJ software v.1.53g (ImageJ 2022; W. Rasband, USA).

### 4.6. Western Blot

Proteins were extracted from HeLa and SH-SY5Y cells using RIPA buffer (150 mM sodium chloride, 1% NP-40, 0.5% sodium deoxycholate, 0.1% sodium dodecyl sulphate (SDS), 50 mM Tris, pH 8.0) and quantified by BCA assay (Sigma-Aldrich, St. Louis, MO, USA). Protein quantity and quality were assessed using a NanoDrop spectrophotometer (ThermoFisher Scientific, Waltham, MA, USA).

WB analysis was performed by SDS-polyacrylamide gel electrophoresis (SDS-PAGE). In total, 50 µg of proteins was loaded into a 12.5% SDS-PAGE gel and, after electrophoresis, the gel was transferred to nitrocellulose membrane (BioRad, Hercules, CA, USA). Membranes were blocked in 5% non-fat dry milk diluted in 1X TBS-T buffer (10 mM Tris-HCl, 100 mM NaCl, 0.1% Tween, pH 7.5) and incubated in primary antibody overnight at 4 °C (Table S3). The next day, the immunoreactivity was detected using secondary peroxidase-conjugated antibodies (Table S3) and visualized using an ECL chemiluminescence detection kit (Sigma-Aldrich, St. Louis, MO, USA). Densitometric analysis was performed using ImageJ software v.1.53g (ImageJ 2022; W. Rasband, USA).

### 4.7. RNA Extraction

RNA from PBMCs was extracted using Trizol^®^ (Invitrogen, Waltham, MA, USA) and following the manufacturer’s instructions. RNA quantity and quality were assessed using a NanoDrop spectrophotometer and Qubit device (ThermoFisher Scientific, Waltham, MA, USA). RNA purity was evaluated by loading 500 ng of RNA into a 1% Agarose gel with Ethidium Bromide (ThermoFisher Scientific, Waltham, MA, USA) and running the samples for 35 min at 120 V. The resulting gel was analyzed for the presence of 28S and 18S bands. Samples that showed a degradation of the RNA or a contamination by DNA were excluded from the study. RNA was then reverse-transcribed using the iScriptcDNA Synthesis Kit (BioRad, Hercules, CA, USA) following the manufacturer’s instructions.

### 4.8. 3′ Rapid Amplification of cDNA Ends

In total, 1 μg of total RNA was reverse-transcribed for the first-strand cDNA synthesis with Adapter Primer, performed using SuperScript II RT (Invitrogen, Waltham, MA, USA) and followed by RNaseH treatment. PCR amplifications, with a gene-specific primer (SOD1: GCAGGTCCTCACTTTAATCCTCTATCCAG; GAPDH: TCCCTGAGCTGAACGGGAAG) and the Universal Amplification Primer, were carried out with PfU Ultra II Fusion HS DNA Polymerase (Agilent Technologies, Santa Clara, CA, USA) starting from 2 μg of cDNA and according to the manufacturer’s recommendations. PCR products were separated and analyzed on a 1.5% Agarose gel. *GAPDH* was used as a positive control.

### 4.9. Multiplex Reverse Transcription–Polymerase Chain Reaction

cDNA from SH-SY5Y cells and from patient PBMCs (50 ng) was analyzed by multiplex RT-PCR using the CFX Connect™ Real-Time PCR Detection System (BioRad, Hercules, CA, USA) and following the manufacturer’s instructions. Primers were designed for TOT *SOD1*, for LONG *SOD1*, and for the housekeeping gene *UBC*. The TOT *SOD1* primer was attached to a FAM-BHQ1, whereas the primer for LONG *SOD1* was bound to a TexasRed-BHQ2 fluorophore, and the *UBC* primer to a HEX-BHQ1 probe. Primers for TOT *SOD1*, LONG *SOD1*, and *UBC* are reported in Table S4.

Cycling conditions were set at 95 °C for 3 min, 95 °C for 45 PCR cycles of 10 s each, and 60 °C for 1 min. Cycle threshold (Ct) values were normalized to *UBC*, and the 2ΔCt method was used to evaluate the differences between gene expressions. The ΔCt for SHORT *SOD1* was calculated by subtracting the value of the LONG *SOD1* from that of TOT *SOD1*.

Multiplex RT-PCR values, for TOT *SOD1*, LONG *SOD1*, and SHORT *SOD1* of sALS patients, were correlated with patients’ age at symptom onset, disease severity, and with PRB and PRL.

For the severity of the disease, the ALSFRS_R was used [35]. According to this scale, patients with a lower value of ALSFRS_R were considered to have a higher severity of the pathology [36].

With regard to the progression rate, both PRB and PRL were calculated as described by Kjældgaard et al. (2021), using the following formulas [37]:

$$PRB=\frac{48-(Total\;\text{ALSFRS\_R}\;at\;initial\;visit)}{Symptoms\;duration\;(months)}$$

$$PRL=\frac{48-(Total\;\text{ALSFRS\_R}\;at\;last\;visit)}{Symptoms\;duration\;(months)}$$

### 4.10. Statistical Analysis

Each experiment was performed three times. Statistical analysis was performed using GraphPad Prism (GraphPad Prism 8). Unpaired one-tailed *t*-tests were performed when a comparison between two groups occurred, whereas the ANOVA test followed by the Bonferroni test was used when a comparison between more than two groups occurred. In correlation analysis, the one-tailed Spearman test was performed. *p*-values < 0.05 were considered statistically significant. Data are reported as mean ± S.E.M.

## 5. Conclusions

Although the evidence gathered by this study address some limitations and needs further confirmation, a correlation between *SOD1* mRNAs, especially the SHORT *SOD1* transcript, and ALS diagnosis or prognosis appears promising. These findings have suitable implications for establishing a new potential biomarker for the disease, but also, as indicators of the disease progression rate, they are crucial from the perspective of drug development.

## Data Availability

The datasets used and/or analyzed during the current study are available from the Zenodo repository, 10.5281/zenodo.10066963.

## Supplementary Materials

The supporting information can be downloaded at https://www.mdpi.com/article/10.3390/ijms26146788/s1.

## Abbreviations

The following abbreviations are used in this manuscript:

ALS	Amyotrophic lateral sclerosis
ALSFRS_R	ALS Functional Rating Scale_Revised
Ct	Cycle threshold
CTRL	Control
FLAG	N’terminal-Flag sequence
H_2_O_2_	Hydrogen peroxide
IF	Immunofluorescence
LONG *SOD1*	Long *SOD1* transcript
NT	Not transfected
OS	Oxidative stress
PBMCs	Peripheral blood mononuclear cells
PRB	Basal progression rate
PRL	Late progression rate
RACE	3′ rapid amplification of cDNA ends
RT-PCR	Reverse transcription–polymerase chain reaction
S.E.M.	Standard error of mean
SDS	Sodium dodecyl sulphate
SDS-PAGE	SDS-polyacrylamide gel electrophoresis
SHOR *SOD1*	Short *SOD1* transcript
*SOD1*	Superoxide dismutase 1
TOT *SOD1*	Total *SOD1*
WB	Western blot

## References

[1] Logroscino G., Urso D., Tortelli R. (2022). The challenge of amyotrophic lateral sclerosis descriptive epidemiology: To estimate low incidence rates across complex phenotypes in different geographic areas. Curr. Opin. Neurol..

[2] Niedermeyer S., Murn M., Choi P.J. (2019). Respiratory Failure in Amyotrophic Lateral Sclerosis. Chest.

[3] Dhasmana S., Dhasmana A., Narula A.S., Jaggi M., Yallapu M.M., Chauhan S.C. (2022). The panoramic view of amyotrophic lateral sclerosis: A fatal intricate neurological disorder. Life Sci..

[4] Zou Z.Y., Zhou Z.R., Che C.H., Liu C.Y., He R.L., Huang H.P. (2017). Genetic epidemiology of amyotrophic lateral sclerosis: A systematic review and meta-analysis. J. Neurol. Neurosurg. Psychiatry.

[5] Vidovic M., Müschen L.H., Brakemeier S., Machetanz G., Naumann M., Castro-Gomez S. (2023). Current State and Future Directions in the Diagnosis of Amyotrophic Lateral Sclerosis. Cells.

[6] Norris S.P., Likanje M.N., Andrews J.A. (2020). Amyotrophic lateral sclerosis: Update on clinical management. Curr. Opin. Neurol..

[7] Rosen D.R., Siddique T., Patterson D., Figlewicz D.A., Sapp P., Hentati A., Donaldson D., Goto J., P O J., Deng H.X. (1993). Mutations in Cu/Zn superoxide dismutase gene are associated with familial amyotrophic lateral sclerosis. Nature.

[8] Müller K., Oh K.W., Nordin A., Panthi S., Kim S.H., Nordin F., Freischmidt A., Ludolph A.C., Ki C.S., Forsberg K. (2022). De novo mutations in *SOD1* are a cause of ALS. J. Neurol. Neurosurg. Psychiatry.

[9] Garcia C., Vidal-Taboada J.M., Syriani E., Salvado M., Morales M., Gamez J. (2019). Haplotype Analysis of the First A4V-*SOD1* Spanish Family: Two Separate Founders or a Single Common Founder?. Front. Genet..

[10] Zelko I.N., Mariani T.J., Folz R.J. (2002). Superoxide dismutase multigene family: A comparison of the CuZn-SOD (SOD1), Mn-SOD (SOD2), and EC-SOD (SOD3) gene structures, evolution, and expression. Free Radic. Biol. Med..

[11] Taylor J.P., Brown RHJr Cleveland D.W. (2016). Decoding ALS: From genes to mechanism. Nature.

[12] Fang T., Je G., Pacut P., Keyhanian K., Gao J., Ghasemi M. (2022). Gene Therapy in Amyotrophic Lateral Sclerosis. Cells.

[13] Zhao X., Feng X., Li X., Mou J., Liu H., Chen J., Wu J. (2022). The G41D mutation in SOD1-related amyotrophic lateral sclerosis exhibits phenotypic heterogeneity among individuals: A case report and literature review. Medicine.

[14] Scarian E., Bordoni M., Fantini V., Jacchetti E., Raimondi M.T., Diamanti L., Carelli S., Cereda C., Pansarasa O. (2022). Patients’ Stem Cells Differentiation in a 3D Environment as a Promising Experimental Tool for the Study of Amyotrophic Lateral Sclerosis. Int. J. Mol. Sci..

[15] Pansarasa O., Bordoni M., Diamanti L., Sproviero D., Gagliardi S., Cereda C. (2018). SOD1 in Amyotrophic Lateral Sclerosis: “Ambivalent” Behavior Connected to the Disease. Int. J. Mol. Sci..

[16] Pansarasa O., Garofalo M., Scarian E., Dragoni F., Garau J., Di Gerlando R., Diamanti L., Bordoni M., Gagliardi S. (2022). Biomarkers in Human Peripheral Blood Mononuclear Cells: The State of the Art in Amyotrophic Lateral Sclerosis. Int. J. Mol. Sci..

[17] Grad L.I., Yerbury J.J., Turner B.J., Guest W.C., Pokrishevsky E., O’Neill M.A., Yanai A., Silverman J.M., Zeineddine R., Corcoran L. (2014). Intercellular propagated misfolding of wild-type Cu/Zn superoxide dismutase occurs via exosome-dependent and -independent mechanisms. Proc. Natl. Acad. Sci. USA.

[18] Ayers J.I., Fromholt S.E., O’Neal V.M., Diamond J.H., Borchelt D.R. (2016). Prion-like propagation of mutant SOD1 misfolding and motor neuron disease spread along neuroanatomical pathways. Acta Neuropathol..

[19] Silverman J.M., Christy D., Shyu C.C., Moon K.-M., Fernando S., Gidden Z., Cowan C.M., Ban Y., Stacey R.G., Grad L.I. (2019). CNS-derived extracellular vesicles from superoxide dismutase 1 (SOD1)^G93A^ ALS mice originate from astrocytes and neurons and carry misfolded SOD1. J. Biol. Chem..

[20] Mackenzie I.R., Bigio E.H., Ince P.G., Geser F., Neumann M., Cairns N.J., Kwong L.K., Forman M.S., Ravits J., Stewart H. (2007). Pathological TDP-43 distinguishes sporadic amyotrophic lateral sclerosis from amyotrophic lateral sclerosis with SOD1 mutations. Ann. Neurol..

[21] Benson B.C., Shaw P.J., Azzouz M., Highley J.R., Hautbergue G.M. (2021). Proteinopathies as Hallmarks of Impaired Gene Expression, Proteostasis and Mitochondrial Function in Amyotrophic Lateral Sclerosis. Front. Neurosci..

[22] Sherman L., Levanon D., Lieman-Hurwitz J., Dafni N., Groner Y. (1984). Human Cu/Zn superoxide dismutase gene: Molecular characterization of its two mRNA species. Nucleic Acids Res..

[23] Milani P., Amadio M., Laforenza U., Dell M., Diamanti L., Sardone V., Gagliardi S., Govoni S., Ceroni M., Pascale A. (2013). Posttranscriptional regulation of SOD1 gene expression under oxidative stress: Potential role of ELAV proteins in sporadic ALS. Neurobiol. Dis..

[24] Dell’Orco M., Milani P., Arrigoni L., Pansarasa O., Sardone V., Maffioli E., Polveraccio F., Bordoni M., Diamanti L., Ceroni M. (2016). Hydrogen peroxide-mediated induction of SOD1 gene transcription is independent from Nrf2 in a cellular model of neurodegeneration. Biochim. Biophys. Acta.

[25] Bordoni M., Pansarasa O., Dell’Orco M., Crippa V., Gagliardi S., Sproviero D., Bernuzzi S., Diamanti L., Ceroni M., Tedeschi G. (2019). Nuclear Phospho-SOD1 Protects DNA from Oxidative Stress Damage in Amyotrophic Lateral Sclerosis. J. Clin. Med..

[26] Meiering E.M. (2008). The threat of instability: Neurodegeneration predicted by protein destabilization and aggregation propensity. PLoS Biol..

[27] McAlary L., Plotkin S.S., Yerbury J.J., Cashman N.R. (2019). Prion-Like Propagation of Protein Misfolding and Aggregation in Amyotrophic Lateral Sclerosis. Front. Mol. Neurosci..

[28] Cozzolino M., Pesaresi M.G., Gerbino V., Grosskreutz J., Carrì M.T. (2012). Amyotrophic lateral sclerosis: New insights into underlying molecular mechanisms and opportunities for therapeutic intervention. Antioxid. Redox Signal..

[29] Motataianu A., Serban G., Barcutean L., Balasa R. (2022). Oxidative Stress in Amyotrophic Lateral Sclerosis: Synergy of Genetic and Environmental Factors. Int. J. Mol. Sci..

[30] Pal S., Tiwari A., Sharma K., Sharma S.K. (2020). Does conserved domain SOD1 mutation has any role in ALS severity and therapeutic outcome?. BMC Neurosci..

[31] Berdyński M., Miszta P., Safranow K., Andersen P.M., Morita M., Filipek S., Żekanowski C., Kuźma-Kozakiewicz M. (2022). SOD1 mutations associated with amyotrophic lateral sclerosis analysis of variant severity. Sci. Rep..

[32] Couratier P., Lautrette G., Luna J.A., Corcia P. (2021). Phenotypic variability in amyotrophic lateral sclerosis. Rev. Neurol..

[33] Ludolph A., Drory V., Hardiman O., Nakano I., Ravits J., Robberecht W., Shefner J., WFN Research Group On ALS/MND (2015). A revision of the El Escorial criteria—2015. Amyotroph. Lateral Scler. Front. Degener.

[34] Filosto M., Piccinelli S.C., Palmieri I., Necchini N., Valente M., Zanella I., Biasiotto G., Di Lorenzo D., Cereda C., Padovani A. (2018). A Novel Mutation in the Stalk Domain of *KIF5A* Causes a Slowly Progressive Atypical Motor Syndrome. J. Clin. Med..

[35] Gordon P.H., Miller R.G., Moore D.H. (2004). ALSFRS-R. Amyotroph. Lateral Scler. Other Mot. Neuron Disord..

[36] Cunha-Oliveira T., Montezinho L., Mendes C., Firuzi O., Saso L., Oliveira P.J., Silva F.S.G. (2020). Oxidative Stress in Amyotrophic Lateral Sclerosis: Pathophysiology and Opportunities for Pharmacological Intervention. Oxid. Med. Cell. Longev..

[37] Kjældgaard A.L., Pilely K., Olsen K.S., Lauritsen A.Ø., Pedersen S.W., Svenstrup K., Karlsborg M., Thagesen H., Blaabjerg M., Theódórsdóttir Á. (2021). Complement Profiles in Patients with Amyotrophic Lateral Sclerosis: A Prospective Observational Cohort Study. J. Inflamm. Res..

