# Supplementary material for: Impact of SOD1 Transcript Variants on Amyotrophic Lateral Sclerosis Severity
Source: Int J Mol Sci. 2025 Jul 15;26(14):6788. doi: 10.3390/ijms26146788 (PMC12295590; doi:10.3390/ijms26146788)
Supplement: Supplementary file 1 [file ijms-26-06788-s001.zip › Legend of Supplementary Figures.docx]

**Legend of Supplementary Figures**

Figure S1: The expression of endogenous *SOD1* does not differ in both transfected and not-transfected HeLa cells. Four *SOD1* plasmids were generated and transfected in HeLa cells: CDS *SOD1* (coding sequence only which ends with the stop codon), SHORT *SOD1* (transcript which ends with the 2nd polyA signal), LONG *SOD1* (transcript which terminates with the 4th polyA site) and END *SOD1* (transcript which terminates with the 4th polyA site). NT indicates not-transfected Hela cells. A-D WB analysis for total *SOD1*, transfected *SOD1* and endogenous *SOD1*. A) Total *SOD1* and B) transfected *SOD1* expression confirmed the corrected transfection in HeLa cells. C) No significant differences were found in endogenous *SOD1* expression. D) Representative WB of endogenous (16 kDa) and transfected (18 kDa) protein levels. GAPDH was used as loading control (37 kDa). E-H IF analysis for total SOD1, transfected SOD1 and endogenous SOD1. E-G) No significant differences were found in total SOD1, in transfected SOD1 and in endogenous SOD1 expression. H) Representative images of IF analysis (red= transfected SOD1, green= endogenous SOD1, blue= nuclei stained by DAPI). Error bars indicate S.E.M. N=3. Data were analyzed using ANOVA test followed by Bonferroni test.

Figure S2: PRB value does not correlate with SOD1 transcripts expression. The levels of TOT *SOD1*, LONG *SOD1* and SHORT *SOD1* were correlated with the PRB values. No significant correlations were found for A) TOT *SOD1*, B) LONG *SOD1* and C) SHORT *SOD1*. Data were analyzed using one-tailed Spearman test. N=10.

Figure S3: PRL value does not correlate with *SOD1* transcripts expression. The levels of TOT *SOD1*, LONG *SOD1* and SHORT *SOD1* were correlated with the PRL values. No significant correlations were found for A) TOT *SOD1*, B) LONG *SOD1* and C) SHORT *SOD1*. Data were analyzed using one-tailed Spearman test. N=9.

Figure S4. *SOD1* mRNA complete sequence. The green highlighted base indicates the end of the short mRNA transcript, whereas the red highlighted base represents the end of the long mRNA transcript
