# Supplementary material for: Impact of SOD1 Transcript Variants on Amyotrophic Lateral Sclerosis Severity
Source: Int J Mol Sci. 2025 Jul 15;26(14):6788. doi: 10.3390/ijms26146788 (PMC12295590; doi:10.3390/ijms26146788)
Supplement: Supplementary file 1 [file ijms-26-06788-s001.zip › Table S1.docx]

| **Sample** | **Sex** | **First visit  age** | **Onset site** | **Disease duration  at first visit (months)** | **ALSFRS  at first visit** | **PRB** | **Disease duration  at follow up (months)** | **ALSFRS  at follow up** | **PRL** |
| --- | --- | --- | --- | --- | --- | --- | --- | --- | --- |
|  |  |  |  |  |  |  |  |  |  |
| ALS1 | M | 64 | Bulbar | 25 | 39/48 | 0,36 | 37 | 38/48 | 0,27 |
| ALS2 | M | 62 | Spinal | 6 | 27/48 | 3,50 | 12 | 5/48 | 3,56 |
| ALS3 | F | 67 | Spinal | 5 | 40/48 | 1,60 | 11 | 13/48 | 3,20 |
| ALS4 | M | 82 | Spinal | 12 | 33/48 | 1,25 | 21 | 21/48 | 1,27 |
| ALS5 | M | 65 | Spinal | 22 | 42/48 | 0,27 | 30 | 33/48 | 0,50 |
| ALS6 | F | 62 | Spinal | 9 | 43/48 | 0,56 | 18 | 27/48 | 1,17 |
| ALS7 | M | 57 | Spinal | 2 | 43/48 | 2,50 | 10 | 36/48 | 1,25 |
| ALS8 | F | 57 | Spinal | 40 | 43/48 | 0,13 | 47 | 31/48 | 0,36 |
| ALS9 | F | 81 | Spinal | 40 | 29/48 | 0,13 | 54 | 26/48 | 0,40 |
| ALS10 | M | 78 | Spinal | 48 | 41/48 | 0,15 | 69 | 38/48 | 0,15 |

Table S1. Clinical characterization of sALS patients. PRB: progression rate (basal); PRL: progression rate (late).
