# Supplementary material for: Impact of SOD1 Transcript Variants on Amyotrophic Lateral Sclerosis Severity
Source: Int J Mol Sci. 2025 Jul 15;26(14):6788. doi: 10.3390/ijms26146788 (PMC12295590; doi:10.3390/ijms26146788)
Supplement: Supplementary file 1 [file ijms-26-06788-s001.zip › Table S2.docx]

| **Primary Antibody** | **Secondary Antibody** |
| --- | --- |
| SOD1 1:700  (Santa Cruz Biotechnology, USA) | CFTM 488A goat anti-rabbit 1:350  (Sigma-Aldrich, USA) |
| Anti-FLAG M2 1:700  (Agilent Technologies, USA) | CFTM 594 goat anti-mouse 1:350  (Sigma-Aldrich, USA) |

Table S2. Primary and secondary antibodies used for IF analysis in HeLa and SH-SY5Y cells.
