# Supplementary material for: Impact of SOD1 Transcript Variants on Amyotrophic Lateral Sclerosis Severity
Source: Int J Mol Sci. 2025 Jul 15;26(14):6788. doi: 10.3390/ijms26146788 (PMC12295590; doi:10.3390/ijms26146788)
Supplement: Supplementary file 1 [file ijms-26-06788-s001.zip › Table S3.docx]

| **Primary Antibody** | **Secondary Antibody** |
| --- | --- |
| SOD1 1:1000  (Santa Cruz Biotechnology, USA) | Anti-rabbit 1:8000  peroxidase-conjugated antibody  (Sigma-Aldrich, USA) |
| Anti-FLAG M2 1:1000  (Agilent Technologies, USA) | Anti-mouse 1:5000  peroxidase-conjugated antibody  (Sigma-Aldrich, USA) |

Table S3. Primary and secondary antibodies used for WB analysis in HeLa and SH-SY5Y cells.
