# Supplementary material for: Impact of SOD1 Transcript Variants on Amyotrophic Lateral Sclerosis Severity
Source: Int J Mol Sci. 2025 Jul 15;26(14):6788. doi: 10.3390/ijms26146788 (PMC12295590; doi:10.3390/ijms26146788)
Supplement: Supplementary file 1 [file ijms-26-06788-s001.zip › Table S4.docx]

| **Gene** | **Primer sequences (5’-3’)** |
| --- | --- |
| **TOT *SOD1*** | **F** CCATGTTCATGAGTTTGGAGATAAT  **R** TGCCTCTCTTCATCCTTTGG |
| **LONG *SOD1*** | **F** CTTTAAAGTACCTGTAGTGA GAAAC  **R** TAGCCTCATAATAAGTGCCATACA |
| ***UBC*** | **F** CCACTCTGCACTTGGTCCTG  **R** TGCAACAACTTTATTGAAAGGAAA |

Table S4. List of Multiplex RT-PCR primers for TOT *SOD1*, LONG *SOD1* and *UBC*.
