# Supplementary figures and images for: Impact of SOD1 Transcript Variants on Amyotrophic Lateral Sclerosis Severity
Source: Int J Mol Sci. 2025 Jul 15;26(14):6788. doi: 10.3390/ijms26146788 (PMC12295590; doi:10.3390/ijms26146788)

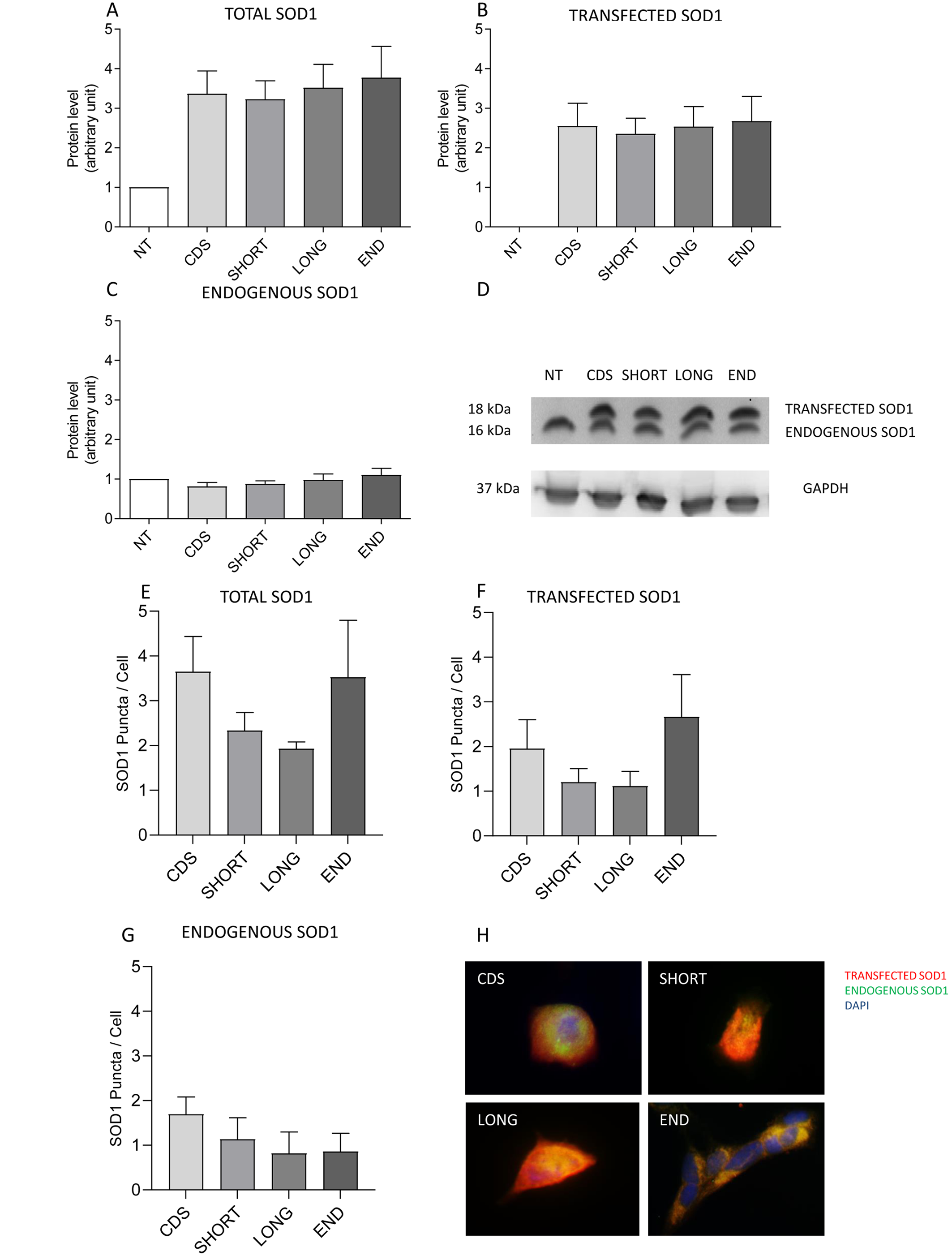

Supplement: Supplementary file 1 [file ijms-26-06788-s001.zip › Figure S1.tif]

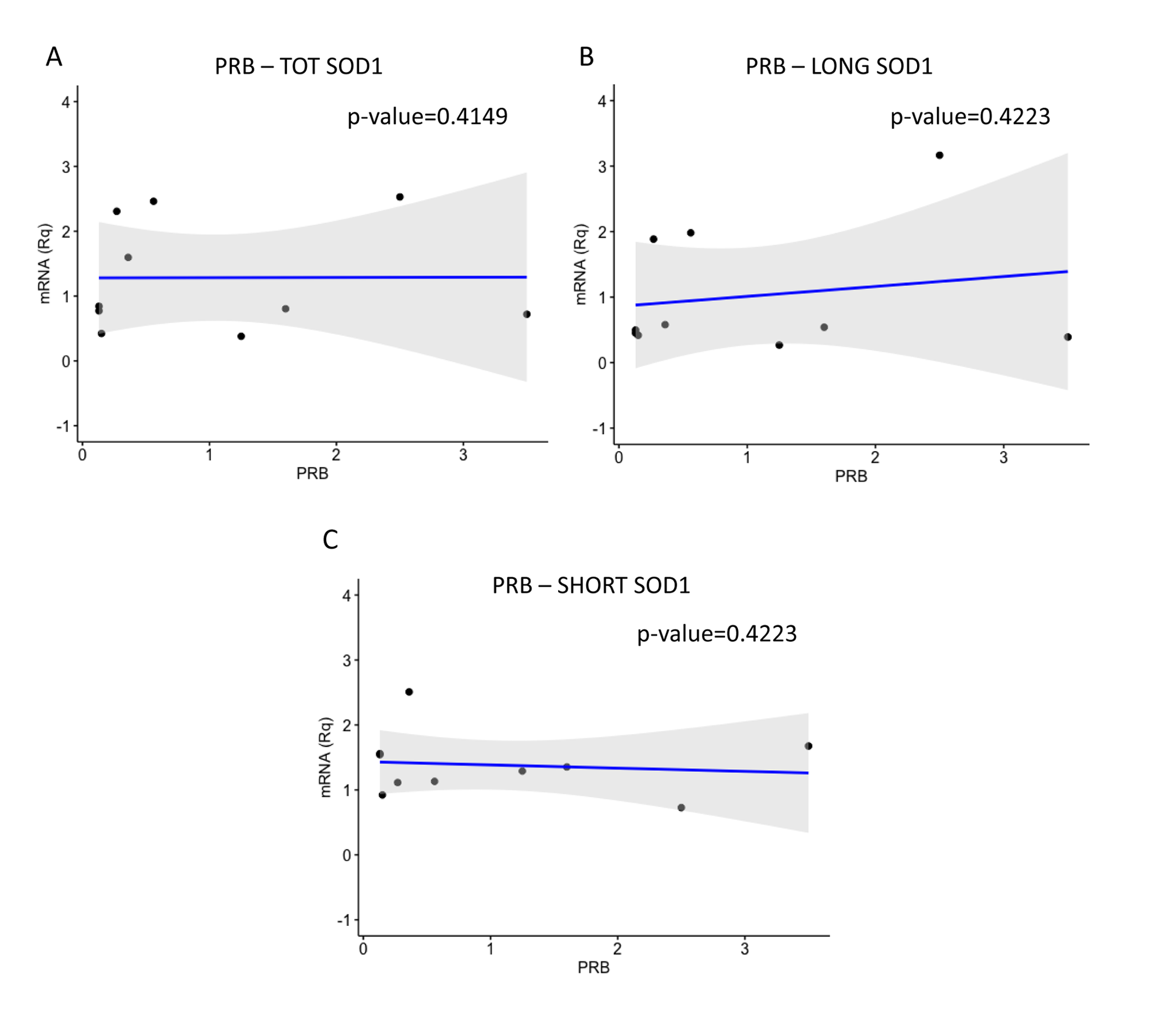

Supplement: Supplementary file 1 [file ijms-26-06788-s001.zip › Figure S2.tif]

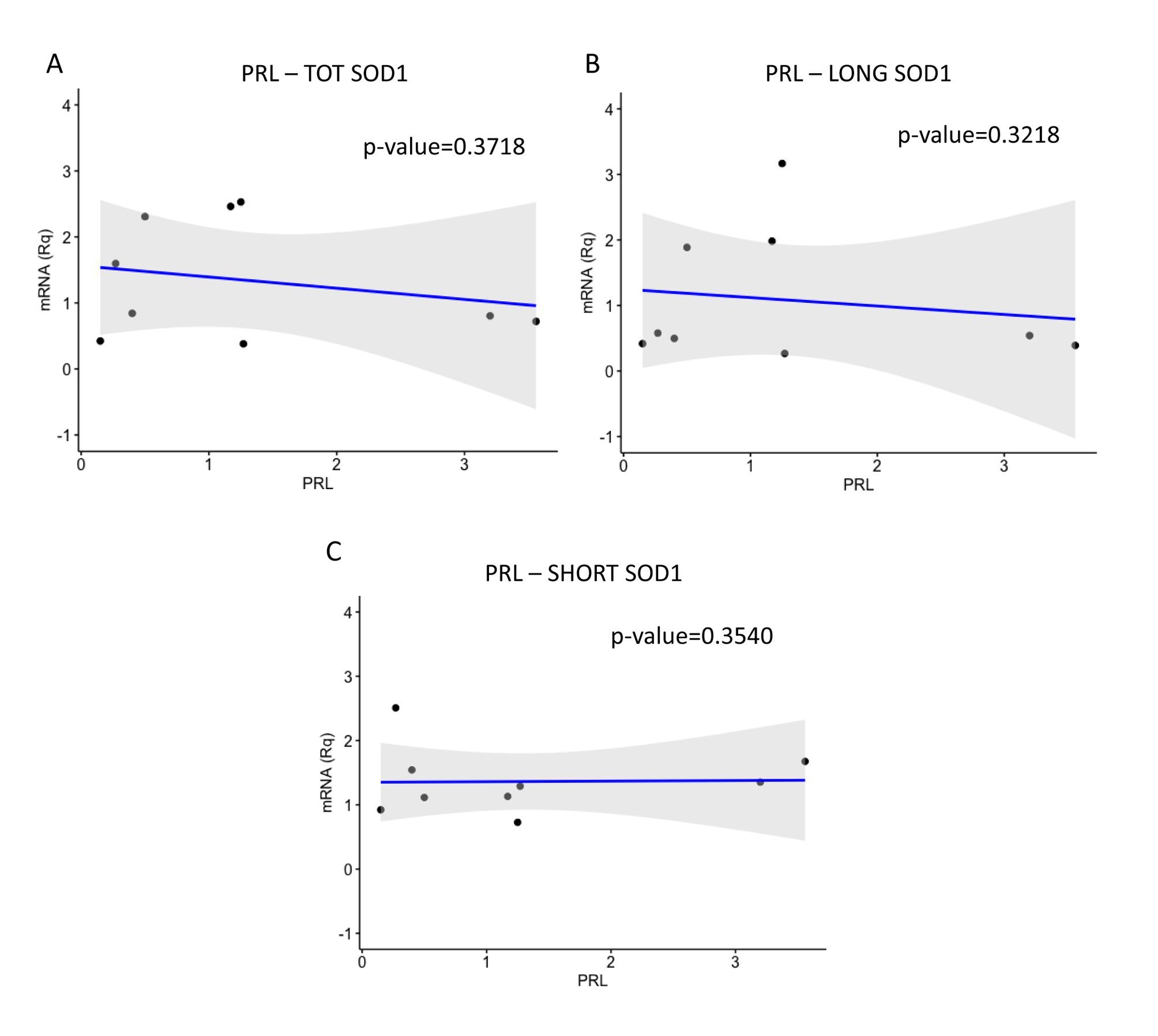

Supplement: Supplementary file 1 [file ijms-26-06788-s001.zip › Figure S3.tif]

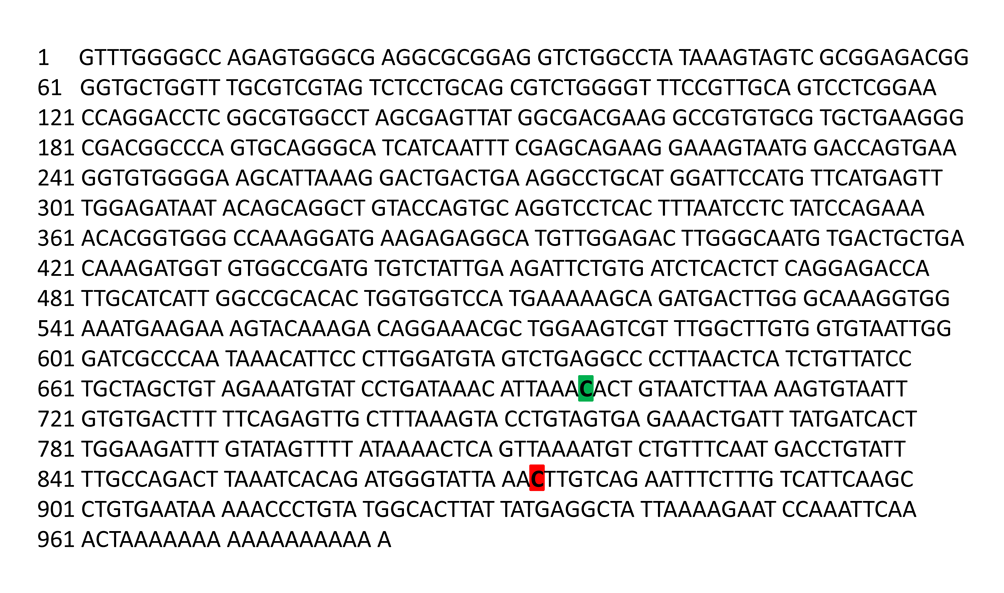

Supplement: Supplementary file 1 [file ijms-26-06788-s001.zip › Figure S4.tif]
